# Family and Peer Support Facilitates Recall and Retelling of Traumatic Memories in War Refugee Children and Adolescents

**DOI:** 10.3390/ijerph22030328

**Published:** 2025-02-23

**Authors:** Arianna Barazzetti, Stefano Milesi, Francesca Giordano, Maria Chiara Noto, Attà Negri

**Affiliations:** 1 Department of Human and Social Sciences, University of Bergamo, 24129 Bergamo, Italy; stefano.milesi@unibg.it (S.M.); atta.negri@unibg.it (A.N.); 2 Milan Centre of Family Therapy, 20123 Milan, Italy; mariachiara.noto01@gmail.com; 3 Resilience Research Unit, University Catholic of Milan, 20123 Milan, Italy; francesca.giordano@unicatt.it

**Keywords:** war refugees, war trauma, PTSD, resilience, meaning making, narrative exposure therapy, family and peer support, dissociation, trauma memory

## Abstract

War refugees are more likely to present psychological disorders, such as PTSD. Memory impairments often hinder their ability to recall and integrate traumatic events. This study investigated the memory capacity of 133 Syrian refugee children and adolescents in Lebanon and examined the moderating role of resilience in the relationship between exposure to negative events, post-traumatic stress reactions, and traumatic events recollection. Participants completed the Childhood War Trauma Questionnaire (CWTQ), Post-Traumatic Symptom Reactions Checklist for Children (PTSR-C), and Child and Youth Resilience Measure-28 (CYRM-28). They also performed a timeline exercise, part of Narrative Exposure Therapy, in which they organized and described their positive and negative life memories. Results showed that memories of traumatic events were lower than potentially traumatic events that participants had witnessed. This difference increased as post-traumatic stress increased. Moreover, potentially traumatic events experienced predicted post-traumatic stress reactions, and these predicted negative memories recalled. Participants’ resilience moderated the association between post-traumatic reactions and remembered traumatic memories showing that only for children and adolescents with higher resilience post-traumatic symptoms predicted trauma-related memories. The results highlight meaning making and sharing capacities of natural relational contexts (family, peers, etc.) as potential key processes to be promoted to overcome and process traumatic events.

## 1. Introduction

The long civil war that began in Syria in 2011 has caused a series of mass emigrations from the country [1]. More than five million Syrian citizens have left their homeland; children account for about half of the refugees, and nearly three-quarters of them were under the age of 12 [2]. The main destination for Syrian refugees has been Syria’s neighboring countries, particularly Turkey, Iraq, Jordan, and Lebanon. In 2015, there were 1,113,941 registered Syrian refugees in Lebanon, of whom it is estimated that 78% were children and women [3,4].

The adverse impact of traumatic events on mental health among refugees has been extensively documented, especially for children and adolescents. Epidemiological surveys conducted among refugee populations have revealed several maladaptive symptoms and the post-traumatic stress disorder (PTSD) emerging as the most prevalent disorder [5,6]. Prevalence rates among refugee children for PTSD range from 19 to 54 percent and for depression from 3 to 30 percent, but many other emotional and behavioral difficulties are well documented [7,8,9,10]. Prolonged economic hardship, injustice and violence experienced in home countries, resettlement in another country, bereavement due to war and emigration, separation of family members, loss of ties to home and community, and important differences between home and host country education systems heavily influence the mental health of war refugee children [11,12].

Numerous studies have explored the association between war atrocities, refugee conditions, and psychosocial adaptation issues [4,13]. However, much remains to be investigated regarding risk and protective factors in these vulnerability conditions. In fact, the type of traumatic events experienced and their severity can have different effects that in turn can be influenced by many contextual and personal factors. For example, some recent studies explored the influence of gender and age, suggesting that female gender and older age may increase vulnerability to psychosocial maladjustment and negatively impact the mental health outcomes of refugee children and adolescents [11,14].

In the present study, we focused on Syrian refugee children and adolescents in Lebanon. Regarding this population specifically, some studies have documented a wide range of psychosocial and behavioral problems [15,16,17,18]. Cartwright and colleagues, for example, found that nearly half of Syrian refugee children in Turkey manifested clinically significant levels of anxiety and social withdrawal [17]. Another recent study on Syrian refugee children in Lebanon and Jordan documented that 45.6 percent of the sample exposed to war trauma developed PTSD [4,9,19].

### 1.1. Trauma and Memory

Memory impairments associated with post-traumatic stress have been intensively studied and documented in the literature. The overwhelming emotion activation associated with traumatic events could lead to dissociative phenomena that partially and indirectly protect victims from the acute pain triggered by the events [20,21]. These protective phenomena explain two aspects of the process of remembering traumatic events that are seemingly opposite to each other but are both outcomes of the dissociative processes that the traumatic events often activate: on the one hand, the reduction, alteration, or incompleteness of the memory of traumatic events, and on the other hand, the persistence and stability in the memory of certain and partial aspects of traumatic events that became persistent and recurrent.

Individuals who have experienced traumatic events may have difficulty recalling the painful events to which they were exposed, their narrative may be fragmentary or incomplete, and in severe cases, it may be completely impossible to recall the event [22]. Sexually abused children presenting PTSD have poorer recall skills than children of the same age who have not experienced trauma, and they show an increased risk of incorporating incorrect and misleading information after the event, precisely because of PTSD [23]. People who have experienced trauma often employ defense mechanisms such as avoidance, denial, and dissociation to alter the memory of the traumatic events due to their overwhelming nature [24] and marked decrease or significant alterations in number of reported details was detected in children refugee’s recounting of their trauma [25,26]. Other studies have shown that mnemonic difficulties may not only relate to traumatic events but affect other autobiographical memory data, including ordinary events. Numerous investigations involving individuals traumatized by diverse circumstances, including the traumatic event following experiences of loss [27], war-related experiences [4,28,29,30,31], instances of sexual abuse [32], and severe medical conditions [33], have consistently found a general tendency to generate fewer autobiographical memories.

A second effect of dissociative processes is to make traumatic events more persistent in memory, albeit at a pre-reflexive level and incompletely. Some authors claimed that traumatic memories exhibit a greater degree of stability in memory compared to other types of events [34]. For example, PTSD [35] is characterized by intrusive, distressing, and involuntary memories of traumatic events [36,37] that often have no clear spatial or temporal context and are accompanied by intense emotional reactions and subsequent efforts to avoid them [38,39,40,41]. Krans and colleagues [42] comparing traumatized individuals diagnosed with PTSD or without PTSD found a significantly higher presence of trauma-related memories in the PTSD group than in the traumatized individuals without PTSD. McNally’s study of Vietnam war veterans revealed a high prevalence of war-related traumatic memories in veterans with PTSD symptoms, indicating that they recalled fewer recent memories unrelated to war [43]. McNally suggested that the PTSD group exhibited a “psychological fixation” on the war, and the altered temporal distribution of autobiographical memories suggested that these individuals were, in a sense, trapped in the past. Similar findings were reported in Schönfeld and Ehlers’s study, where the PTSD group exhibited vivid and recurrent trauma memories, with few memories unrelated to trauma [41]. Rubin argued that adults affected by PTSD often perceive traumas as central to their life history [44].

### 1.2. Social and Relational Factors Protecting Against the Negative Effects of Traumatic Events

In the literature, there is a consolidated distinction between Type I Trauma—characterized by acute, catastrophic events—generally associated with PTSD, and Type II Trauma—involving prolonged or repeated relational negative events—generally associated with Complex PTSD or developmental trauma. However, as Howell [45] points out, finding a certain and agreed definition of trauma remains complex and difficult. There are certainly events recognized as certainly more traumatic than others [35], such as exposure to life-threatening situations or sexual violence, but the occurrence and intensity of post-traumatic stress symptoms, such as those listed in PTSD, vary according to several variables. These relate to the nature, timing, and manner in which the traumatic events were witnessed but also the personal functioning, social, and interpersonal context in which the individual lives contribute to making the impact of the events more or less traumatic.

In this study, we adopted an intersubjective [46] rather than orthopedic perspective on trauma. The latter sees traumatic events as dangerous elements that damage the individual’s normal psychological functioning from the outside, while we think that events, even the most threatening ones, are not psychologically traumatic per se, but can become traumatic if they create a violation of the individuals’ core beliefs and if their meaning-making abilities fail to integrate them into their value system and personal history [47,48,49,50]. Since the ability to attribute meaning to events is mainly a function of cultural and interpersonal processes, events that become traumatic are those that reduce the possibilities/potential of a family or society to make sense of such events. It is this function that is impaired and not simply the individual psychological functioning, both when the potential traumatic event comes from outside one’s reference groups (e.g., war, emigration, etc.) and when it is perpetuated by members within one’s own group (abuse, neglect, etc.). The protective and healing factors of the negative effects of trauma thus reside in the ability of the relational and social systems of which the individual is a part to creatively reboot the potentials/capabilities of this meaning making, even in the presence of negative and potentially traumatic events. From this perspective, resilience is a property of human systems that helps their constituent individuals cope with unexpected and potentially traumatic events. Resilience is a dynamic developmental process of positive adaptation in the context of adversity that involves drawing on both internal and external resources at different systemic levels (e.g., individual, relational, community/cultural) [51,52,53]. Resilience, therefore, can play a key role in reducing the development of PTSD symptoms following a traumatic event [54] and, vice versa, PTSD can reduce children’s resilience capabilities when exposed to social pressures and influences [55].

Ecological theories of development argue that the psychosocial adjustment of refugee children and adolescents is shaped by a complex interplay of contextual factors, influenced by both their past experiences and current circumstances [56,57,58]. A recent study conducted on a group of Syrian children displaced in Jordan showed that current supportive relationships and pro-social competences play a key role for the resilience and wellbeing of children [58,59]. Supportive family and school environments are also critical for this adjustment. Research has shown that family cohesion is linked to lower levels of psychological distress and depression among immigrant populations, whereas family separation and reunification can increase stress [60,61,62]. The emotional climate within a family significantly impacts children’s abilities to regulate their emotions, contributing to either healthy emotional development or emotional dysregulation [63,64,65]. From this perspective, the relational aspects of resilience, particularly the collective support needed to overcome challenges, play a fundamental role [66].

### 1.3. KidNet, a Narrative Technique to Cope with Traumatic Events for War Refugee Children and Adolescents

It is widely accepted that the capacity to remember and to engage in constructive dialog with others regarding one’s traumatic experiences represents a fundamental protective factor for individuals who have experienced such events. By recounting and narrating these events, individuals can relieve themselves of an overwhelming burden, often perceived as isolating, and simultaneously integrate these experiences into new and broader narratives about their life [67,68].

Based on this consideration, several types of psychotherapy have been developed for the treatment of PTSD symptoms, focusing on integrating memories of traumatic events into individuals’ autobiographical history. It is noteworthy that the act of recalling and recounting traumatic events with a clinician represents a pivotal aspect of all three forms of therapy that have been recommended by the National Institute for Health and Care Excellence (NICE) for the treatment of PTSD: trauma-focused cognitive behavioral therapy (CBT), eye movement desensitization and reprocessing (EMDR), schema therapy, and supported trauma-focused computerized CBT as a facilitated self-help intervention [69].

In emergency contexts such as war and mass emigration, there are unfortunately not enough human, logistical, and time resources for such traditional psychological interventions to support people who have witnessed the potentially traumatic events that war and emigration entails. Therefore, more manageable and viable intervention strategies have been developed for these situations. One of these is the “KidNET”, a specific type of Narrative Exposure Therapy program aimed at treating refugee children suffering from post-traumatic stress disorder (PTSD) [70]. It typically consists of 4 to 10 sessions, each lasting 90 to 120 min, conducted weekly by trained clinical psychologists. The aim is to help children or adolescents to build a chronological narrative of their entire life, with an emphasis on traumatic experiences. By recalling traumatic memories and integrating them into a coherent life narrative, this program reduces the emotional arousal associated with these memories and then promotes better emotional regulation. KidNET not only helps to alleviate symptoms of PTSD but also promotes resilience and a sense of empowerment, which are key components of emotional wellbeing. Therapists offer various hands-on activities to foster participants’ ability to put into words and coherent narratives the sensory, emotional, physical, and psychological experiences felt during the major events that dot their autobiographical history, especially the most negative or traumatic ones. One of these activities is the Timeline Activity—the task on which our study’s investigation focused—during which participants take different flowers and stones, representing positive and negative events, respectively, and place them along a rope to illustrate each of the most significant events in their lives. Other activities include drawing to represent scenes and objects from traumatic experiences and body positioning reenactment, during which participants show the ways in which they physically positioned their bodies during a traumatic experience. These help participants to process not only the cognitive aspects of their experiences but also the emotions and sensations associated with them, overcoming the dissociation in this way. At the end of KidNET, therapists give participants a written record of the narrative reconstruction that occurred during the sessions. This final stage provides participants with a tangible artifact of their resilience and progress. When, in the future, participants reread this record it can have a lasting effect and thus reinforce the emotional and cognitive gains made through therapy.

One of the main advantages of KidNET therapy is its high adaptability to any cultural context and to emergency settings, where human and material resources for more structured interventions are lacking. Moreover, KidNET therapy has received evidence supporting its effectiveness in reducing PTSD symptoms, depression, and suicidal ideation of children who experienced severe war trauma [20,71,72,73,74,75].

### 1.4. Purpose of the Present Study

The main purpose that guided the present study was to investigate the relationship between potentially traumatic events experienced, relational and social resilience factors, post-traumatic stress reactions, and the ability to recall and recount such events (traumatic event memories). To this end, we conducted an empirical study within the activities conducted in support of war refugee children and adolescents in Lebanon. One of these was the KidNET treatment that included the Timeline Activity, considered in this study as a measure of the ability to recall traumatic events in a supportive context. In addition, three other instruments were applied: one (CWTQ) to detect potentially negative events that refugee children and adolescents may have witnessed; one (CYRM-28) to detect participants’ personal, family, and relational resilience resources; and one (PTSR-C) to measure post-traumatic stress reactions.

Our main hypotheses concerned the beneficial role of children’s and adolescents’ resilience resources: We expected that they would reduce the impact of potentially negative events on post-traumatic stress symptomatology and increase the likelihood of recounting traumatic events during KidNET therapy. We hypothesized in fact that one of the ways in which positive and supportive relationships play a protective role is through enhancing the individuals’ propensity to recall and narrate their painful memories. We expected that supportive relational contexts foster trust that other people can be supportive and be potential sources of meaning-making about negative events [76]. As a result, individuals would become more inclined to recall and share information about traumatic experiences, not only to their significant others but also in any context that offers a sense of psychological support. From this perspective, contextual resilience factors would work as a protective factor by facilitating the retelling of trauma narratives with others. As highlighted in the literature, narrative sharing of this kind promotes the integration of traumatic events into the individuals’ narratives of themselves and their lives [67,68].

More specifically, our assumptions were as follows:

(a) Not all potentially traumatic events experienced by participants, registered by CWTQ, would be recalled in the Timeline Activity; the underlying assumption was that the traumatic valence of the events is a function of the meaning attributed to them by those who witnessed them and are also influenced by contextual factors. Thus, the events recalled as traumatic would be fewer than those potentially traumatic experienced by participants.
(b) The difference between the number of potentially traumatic events experienced by the participants (recorded by CWTQ) and the traumatic events recalled in the Timeline Activity would have increased with participants’ increasing post-traumatic stress reactions (detected by PTSR-C); the hypothesis behind this prediction was that post-traumatic reactions are caused by dissociative processes that, among other things, reduce the efficiency of remembering events.
(c) As the number of potentially traumatic events in the participants’ lives increased (recorded by CWTQ), post-traumatic stress reactions (detected by PTSR-C), and events remembered as traumatic in the Timeline Activity would also have increased, as documented in the literature.
(d) As the number of post-traumatic stress reactions increased (detected by PTSR-C), the number of traumatic event memories recollected in the Timeline Activity would also increase. Traumatic memories are in fact generally considered a part of the post-traumatic symptoms.
(e) Higher levels of resilience, as measured by the CYRM-28, would be associated with lower levels of post-traumatic stress reactions as detected by PTSR-C; in fact, numerous studies have shown that resilience is a protective factor that makes negative events less impactful.
(f) Higher levels of resilience, as measured by CYRM-28, would be associated with more traumatic events remembered in the Timeline Activity; these predictions were based on the hypothesis that resilience would reduce dissociative processes that negatively affect the recollection of events.

## 2. Materials and Methods

### 2.1. Participants

The study involved 133 Syrian children and adolescents (age: M = 10.9, SD = 2.03; range = 7–16; gender: F = 54.1%, M = 45.9%) who had experienced at least one traumatic event in their life according to the Childhood War Trauma Questionnaire [77]. All the participants were refugees in Lebanon due to the armed conflict in their home country. At the time of the data collection (July–August 2019), most participants were living in temporary shelters (61%), others in tent settlements (26%), and a minority in regular houses (13%); 3% of them had been in Lebanon less than one year, 79% between one and two years, and 18% for more than two years.

### 2.2. Procedure

Data were collected between July and August 2019 by two psychologists employed in nonprofit agencies in four different Lebanese cities hosting Syrian refugees. These psychologists were Lebanese professionals who shared the same language as the participants, ensuring direct communication in Arabic, the children’s native language. This eliminated the need for translation and facilitated authentic interactions, which were critical to understanding the children’s narratives and experiences.

Participation in the study was completely voluntary, and informed consent was obtained from the participants and their parents prior to the start of the study. The standardized tests were administered under the supervision of the psychologists in sessions of about 2.5 h, attended by participants in groups of approximately 20 children or adolescents of similar age. The study was approved by the IRB of the Catholic University of Milan.

### 2.3. Measures

After presenting the research project, a psychologist proposed the following tests and activities to participants:

- *Childhood War Trauma Questionnaire* (CWTQ) [77]. It is a questionnaire administered as an interview developed to collect socio-demographic information about child history and the type of traumatic events to which the child has been exposed. In the CWTQ, about 45 different types of war-related traumas are listed, divided into the following categorizations: separation, victim of violent acts, involvement in hostilities, displacement, bereavement, exposure to shelling or combat, witness violent acts, physical injuries, emigration, and extreme deprivation.
- *Post-Traumatic Stress Reaction Checklist for Children* (PTSR-C) [78]. This consists of a structured interview for children, focusing on PTSD symptoms that can be associated with war traumatic events. It consists of 14 items that assess the presence or absence of some major PTSD reactions (experiencing a traumatic event, avoidance of or decreased engagement with their environment, and increased alertness). The reliability of PTSR-C in this study was excellent, α = 0.837.
- *Childhood and Youth Resilience Measure-28* (CYRM-28) [53]. This is a 28-item self-report questionnaire developed to assess resilience in children and adolescents. The measure provides a total score of resilience and other scores on eight subscales: personal skills; peer support; social skills; physical support received in the family; psychological support received in the family; and spiritual, educational, and cultural resilience factors. The reliability coefficient for CYRM-28 calculated using this study’s data was excellent, α = 0.898.
- *Timeline Activity* [79]. In this activity, individuals are presented with a rope along with some stones and flowers; they are told that the rope represents the passage of time in their lives, with the event of their birth at one end of the rope and the present moment at the other. They are then asked to arrange and describe their most important memories along the rope, using stones to represent negative memories and flowers to represent positive ones. This activity is one of the key exercises used in KidNET therapy to promote self-awareness of one’s life. Memories recalled are categorized as positive or negative memories by participants; it depends on whether the child represents them on the timeline with a stone (negative) or a flower (positive). For this study, only the negative memories were further distinguished into ‘trauma-related’ and ‘trauma-unrelated’ by the psychologists who conducted the Timeline Activity.

### 2.4. Statistical Analyses

All analyses were performed using the JAMOVI software (version 1.6.16), and the *Medmod* supplementary package for moderation analyses. The analysis plan involved four steps.

First, to test if there were significant differences between male and female participants in the variables considered, we applied a series of Student’s *t*-tests.

Second, the associations between potentially traumatic events experienced (CWTQ), post-traumatic reactions (PTSR-C), resilience (CYRM-28), and memories recalled (total, positive, negative, trauma-related, trauma-unrelated) were tested through a series of Spearman’s rank correlation coefficients analyses. The choice of this type of statistic was guided by the results of Shapiro–Wilk tests that did not show a normal distribution ib the considered variables.

Third, we tested five linear regression models, using resilience as a moderator (CYRM-28), on the associations that were significant at the second step: CWTQ > PTSR-C; CWTQ > trauma-related memories; CWTQ > trauma-unrelated memories; PTSR-C > trauma-related memories; PTSR-C > trauma-unrelated memories.

Finally, we tested in the moderation models found to be significant in the third step the moderating role of resilience subdimensions (CYRM-28)

## 3. Results

### 3.1. Participants’ Level of Psychological Distress

The descriptive statistics about the variables measured through the applied instruments confirmed the high level of psychological suffering of the participants involved. The average number of traumatic events reported by children and adolescents in the CWTQ interview was 7.44 (SD = 5.56). During the Timeline Activity, the children reported an average of 1.77 (SD = 0.78) positive memories and 1.89 (SD = 0.65) negative memories. Out of the negative ones, on average, 0.97 (SD = 0.67) were related to traumatic events, while 0.92 (SD = 0.71) were related to non-traumatic events, as classified by the psychologist. These data seem to support hypothesis (a), in that the number of traumatic event memories (Timeline Activity) is systematically lower (*t* = 13.8, *p* < 0.001) than the number of potentially negative events that they had witnessed (CWTQ).

The average score of the participants’ post-traumatic reactions, as measured by the PTSR checklist, was 5.96 (SD = 5.12). The children’s average score on the CYRM-28 resilience questionnaire was 126 (SD = 9.34).

### 3.2. Gender Differences

When comparing male and female children, no differences were found in post-traumatic symptomatology (PTSR-C) and in the resilience scores (CYRM-28), while the average number of traumatic events experienced by female children was significantly lower than that of male children (CWTQ: *t* = 3.65, *df* = 131, *p* = < 0.001, respectively, 5.89 and 9.26). However, the average number of memories recalled and represented during the Timeline Activity was similar in male and female children, both in total and when differentiated into positive and negative memories or negative trauma-related and negative trauma-unrelated memories.

### 3.3. Association Between Traumatic Events, Resilience, Post-Traumatic Reactions, and Event Memories

To test hypothesis (b), we calculated the difference between CWTQ scores and the number of trauma-related memories recalled during the Timeline Activity (CWTQ—traumatic memories delta score). These difference scores were found to correlate with post-traumatic stress reaction (PTSR-C) scores (*ρ* = 0.318, *p* < 0.001), as hypothesized.

The number of traumatic events experienced by participants and registered through the CWTQ by psychologists positively correlated with the post-traumatic reactions scores (PTSR-C; *ρ* = 0.354, *p* < 0.001) and with trauma-related memories (*ρ* = 0.357, *p* < 0.001) and was negatively correlated with trauma-unrelated memories (*ρ* = −0.231, *p* < 0.008) recalled during the Timeline Activity (see Table 1). These findings support hypothesis (c).

Participants’ post-traumatic stress reaction scores (PTSR-C) did not correlate with the number of positive (flowers) and negative memories (stones) recalled and recounted during the Timeline Activity; however, when considering only the negative memories separated in trauma-related and trauma-unrelated groups, participants’ post-traumatic stress reaction scores (PTSR-C) correlated positively with the former (*ρ* = 0.340, *p* < 0.001) and negatively with latter (*ρ* = −0.308, *p* < 0.001) (see Table 1). Thus, hypothesis (d) is supported.

Resilience as measured by CYRM-28 was not found to be associated either with the number of the traumatic events in the participants’ lives, with post-traumatic reactions, or with the total number of recalled memories (see Table 1). These data do not support hypotheses (e) and (f).

In short, the more frequent the children’s traumatic life events, the higher their post-traumatic reactions and trauma-related memories.

### 3.4. The Moderating Role of Resilience

**Figure 1:**
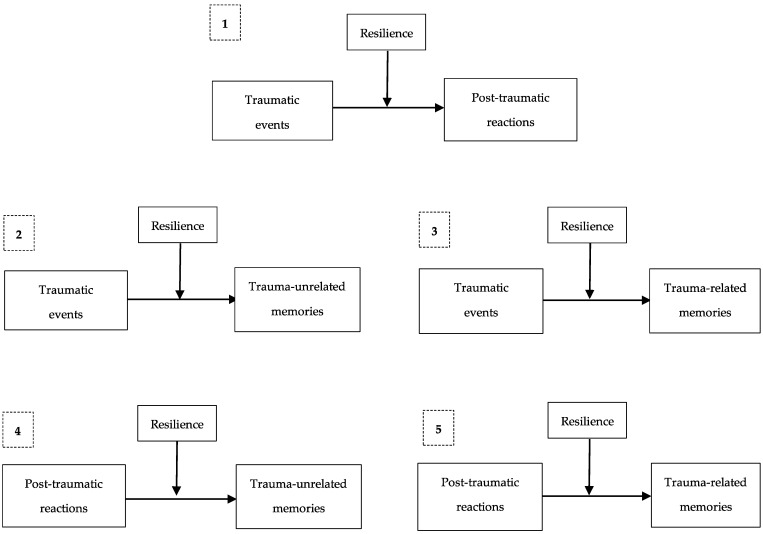
Moderation models tested.

Correlation analysis between the variables (see Table 1) shows there is an association between traumatic life events (CWTQ), post-traumatic reactions (PTSR-C), and recall of trauma-related and trauma-unrelated events, but not between all these variables and resilience (CYRM-28). We tested whether resilience had a moderating role in the associations found in the correlation analysis, i.e., in the relationship between experienced traumatic events and post-traumatic reactions, between experienced traumatic events and trauma-related or trauma-unrelated memories, and between post-traumatic reactions and trauma-related or trauma-unrelated memories (See Figure 1).

In the first three models, the moderation role of resilience (CYRM-28) was not found to be significant: the number of traumatic events experienced (CWTQ-C) predicted post-traumatic reactions scores (PTSR-C) and trauma-unrelated and trauma-related memories (respectively: *R*^2^ = 0.088, *t* = 3.56, *p* < 0.001; *R*^2^ = 0.095, *t* = −2.70, *p* = 0.008; *R*^2^ = 0.095, *t* = 3.72, *p* < 0.001) but resilience did not moderated the impact of these relationships.

Also, in the fourth model, resilience did not have a significant moderating role: the number of trauma-unrelated memories was negatively predicted by participants’ post-traumatic reactions (PTSR-C) (*R*^2^ = 0.091, *t* = −3.63, *p* < 0.001), regardless of resilience level (CYRM-28).

**Figure 2:**
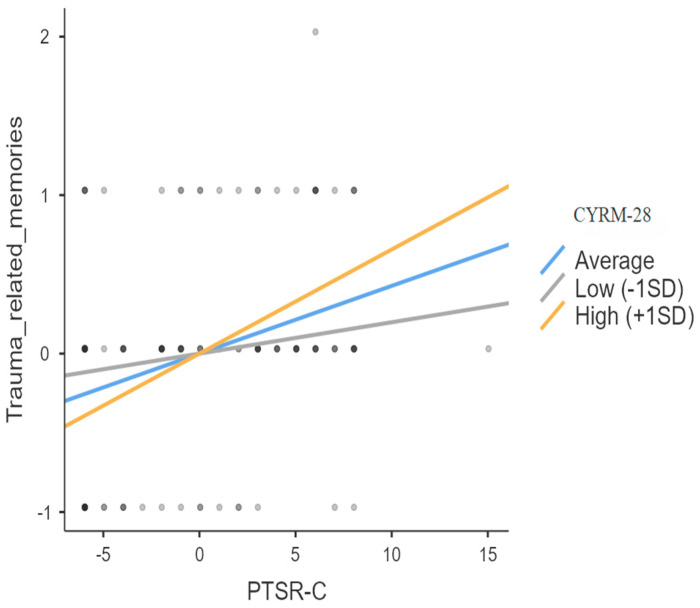
Sample slope plot for the moderation model of resilience (CYRM-28) on the relationship between post-traumatic reaction (PSTR-C) and trauma-related memories.

The last moderation model was found to be significant. In this case, the regression model indicates that post-traumatic stress reactions (PTSR-C) predicted trauma-related memories recalled (*R*^2^ = 0.113, *t* = 4.08, *p* < 0.001), and this relationship is moderated by resilience (CYRM-28) (interaction effect between PTSRC and CYRM-28: *Z* = 2.09, *p* = 0.036). More specifically, the number of trauma-related memories was directly proportional to post-traumatic reactions in the group of children with higher resilience scores (+1 SD: *Z* = 4.29, *p* < 0.001). Conversely, post-traumatic reactions did not significantly predict the recall of traumatic memories in the group of children with lower resilience (−1 SD: *Z* = 1.28, *p* = 0.200) (See Figure 2).

These findings indicate that post-traumatic stress reactions predicted participants’ trauma-related memories only if they had demonstrated high levels of resilience. This result partially supports hypothesis (f), in that the favorable influence of resilience on on traumatic memories works when the level of post-traumatic stress experienced is high.

To deepen this moderating role of resilience, we tested the same moderation model considering each resilience subscales separately in place of the total score (See Table 2). The only subscales that interacted significantly with the PTSR-C scores in predicting the number of trauma related memories were the scales of family psychological support and peer support.

**Figure 3:**
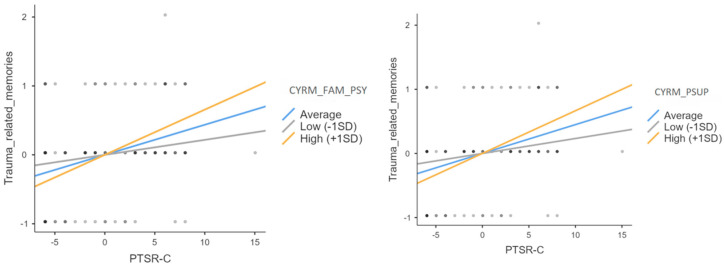
Sample slope plots for the moderation models of Psychological Family Support (CYRM_FAM_PSY) and Peer Support (CYRM_PSUP) subscales on the relationship between post-traumatic reaction (PSTR-C) and trauma-related memories.

With respect to these two subscales that played a significant moderating role, we see that only in children who received higher levels of family support (+1 SD) did a high post-traumatic reaction score (PTSR-C) lead to recounting more trauma-related memories during the Timeline Activity (*Z* = 4.52, *p* < 0.001). In contrast, in children with low family psychological support, the scores of post-traumatic reactions did not predict the ability to recall traumatic memories (*Z* = 1.35, *p* = 0.18) (See Figure 3). Moreover, only in children who received high peer support (+1 SD) did more post-traumatic reactions predict more trauma-related memories recalled *(Z* = 4.23, *p* < 0.001), while for children with low peer support (−1 SD), post-traumatic stress severity did not make the Timeline Activity richer in trauma-related memories (*Z* = 1.55, *p* = 0.12) (See Figure 3).

## 4. Discussion

The CWTQ scores measuring the potentially negative events experienced during war and migration highlight an important level of trauma exposure for children and adolescents participating in this study. These findings confirm once again the huge negative impact of war-related trauma, manifesting as post-traumatic stress symptoms as found in several studies [80,81,82] and documented in the report about Syrian refugees issued by the Eastern Mediterranean Public Health Network [83]. Moreover, our results support the evidence that there are no differences between males and females in the level of post-traumatic reactions experienced. This finding aligns with some studies suggesting that gender differences in PTSD symptoms may not always emerge, particularly when trauma exposure is similar across groups [84,85,86].

As for the specific hypotheses we had made, the study provides substantial confirmation regarding the relationship between potentially traumatic events, post-traumatic stress reactions, and remembered traumatic events, while we received only a partial confirmation regarding the relationship between resilience, post-traumatic symptoms, and recollection of traumatic events. This last unexpected result helps us to better understand how that relationship works.

In our opinion, the difference between the high number of potentially traumatic events recorded by the psychologist through the CWTQ and the significantly lower number of trauma-related events recalled in the Timeline Activity—hypothesis (a)—confirms the important role of the meaning making function of human beings that makes an event traumatic or not because of the meaning attributed to it by those who experience it. As the literature now consistently argues [47,48,49,50], events are not traumatic in themselves but are traumatic to the extent that they violate the basic beliefs of the people who experience them and according to how much these people are able to make sense of such events by integrating them into their own belief and value systems and personal histories.

In addition to the meaning-making function, other factors such as the level of trust established with the psychologist, cultural norms and values, avoidance or dissociative phenomena can have increased the gap between potentially traumatic events and their recollection as such. In any case, the welcoming and facilitating environment created by the psychologists during the Timeline Activity should have limited these effects as much as possible and uniformly for all participants. However, the specific role of dissociative processes commonly associated with traumatic experience was found to increase this gap—hypothesis (b). Such dissociative effects lead to the development of post-traumatic stress reactions and precisely because of their nature they tend to decrease the effectiveness of remembering traumatic events both in terms of the amount of memory recollection and the amount and clarity of remembered details. The positive correlation between post-traumatic stress reactions and the CWTQ-traumatic memories delta scores confirms this hypothesis and supports the assumptions of Salvatore and colleagues who conceive trauma as a narrowing of the interpretative effective range that the relational systems in which individuals are embedded develop to make sense of events [46]. Such narrowing is the outcome of dissociation processes that exclude some meanings from the shared ideational flow and reinforce only some by polarizing them and making them stable and pervasive. For this reason, despite being less detailed and consistent, traumatic memories have a greater impact and significance compared to non-trauma-related memories [87,88].

Regarding the relationships between the level of exposure to potentially traumatic events, post-traumatic stress reactions, and memories of traumatic events, the hypotheses were all confirmed by the results, consistent with findings in the literature [34,36,41,42]. In fact, the number of potentially traumatic events recorded through the CWTQ by psychologists positively correlated with the number of participants’ post-traumatic stress reactions—hypothesis c—as well as the latter positively correlated with the number of negative memories recalled, whether they were trauma-related or trauma-unrelated—hypothesis d.

Noteworthy is the fact that the assumptions we had made about resilience based on the literature were not confirmed by the results of this study. These unexpected results might partially depend on the characteristics of the sample, the tools used, or an interaction between these factors. However, we propose other possible explanations of these missing results that we find very heuristically informative.

First, we did not find that higher levels of resilience were associated with lower levels of post-traumatic stress reactions among study participants—hypothesis (e). On the contrary, the literature emphasizes a complex and negative association between resilience and post-traumatic symptoms [89]. An alternative possible explanation of this missing relationship, in agreement with Bucci [90,91], who highlights the symbolization function performed by the symptoms, could be that post-traumatic stress reactions are the first way to make pain related to the partly dissociated traumatic memories thinkable and communicable rather than being a sign of altered functioning of the individual. In other words, post-traumatic reactions, rather than being diminished by the personal and relational resilience of individuals, are vehicles of communication around which resilient relational contexts activate processes of conversation and resignification that lead to trauma overcoming.

Second, the results did not confirm that higher levels of resilience are associated with higher numbers of traumatic events remembered—hypothesis (f). We assumed this based on the idea that resilience would reduce dissociative processes, which in turn would reduce the effectiveness of remembering events. In part, the findings around hypothesis (e) explain this result because resilience does not actually reduce post-traumatic stress reactions or the indirectly dissociative processes on which they are based. However, the only moderation model that was found to be significant in terms of resilience indicates that resilience (especially the subscales of family and peer psychological support), if present at high levels, has a facilitating role in traumatic event recall and narration in individuals who exhibit high levels of post-traumatic stress reactions.

In other words, our interpretation of these unexpected results is that the participants who seemed to have coped better with the potentially traumatic events were those who relied on higher levels of personal and relational resilience, and this fostered a greater expression of post-traumatic stress reactions that were, in turn, an opportunity to activate conversations in which they shared and recounted the events they experienced as traumatic, thus creating more opportunities to make sense of these events and to functionally integrate them within their own personal history.

All these findings indicate that the processes of meaning-making and the sharing of negative events within one’s support networks (family, peers, etc.) are critical to the psychological elaboration of traumatic events. The creation of therapeutic contexts that ensure and support these processes is essential to obtain good results in terms of reducing the negative impact of traumatic events and in terms of re-signifying them as elements that relaunch one’s future personal development. Not only does therapy need to become a supportive context for these processes, but all psychological interventions should aim to involve and activate the individual’s natural systems of reference (family, peers, school, etc.), even including them in the intervention itself, as in the timeline activity, to reboot and enhance these processes and capacities that are an inherent potential of every human relationship context [92,93].

The study results are insightful, but their generalizability is limited due to the relatively small number of participants in this study; this reduced the statistical power of the analyses and their ability to detect subtle but meaningful interactions. Furthermore, the cross-sectional design of our study presents a challenge in establishing causality between variables. Future longitudinal studies would allow a more in-depth exploration of the complex relationships between negative events, resilience, post-traumatic stress reactions, and memories over time.

Another limitation concerns the tools used, particularly the instrument used to measure resilience. The CYRM-28 captures specific aspects of resilience and conceptualizes it more in contextual and relational rather than individual terms. Other aspects of resilience may certainly have been undetected by this instrument, and this may explain, as mentioned, that the results do not confirm the relationship between resilience and reduction in post-traumatic stress symptoms documented in the literature. Therefore, for a more complete analysis of this relationship, it is desirable that future studies combine more measurement tools of resilience, which is known to be a complex and multidimensional construct.

Also, the data collection period, which took place between July and August 2019, could indicate a further caveat in interpreting the results. The study provide a detailed and meaningful snapshot of the experiences and psychological functioning of Syrian children in that historical moment, but the time between the exile from Lebanon and the survey conducted through the study may have influenced these experiences; socio-cultural dynamics may have evolved over the years, and for instance, an increased exposure to social media and global influences could have reshaped how children narrate traumatic experiences and develop resilience skills.

Furthermore, although KidNET therapy appears to be a useful tool for facilitating the recollection of traumatic events and their integration into personal history, the study does not allow us to comment on its effectiveness or its incremental validity compared to other types of treatment. However, given the extreme negative impact of traumatic events on the mental health of refugees and given their increasing numbers in the today world, it would be useful and urgent to conduct comparative efficacy studies on therapeutic interventions designed to address the specific needs of refugee children.

Despite these limitations, in our opinion, these findings remain relevant as they highlight psychological mechanisms and processes related to trauma and resilience that are likely applicable to similar contexts and populations. The results are novel proof of the variability in how individuals assign meaning to the traumatic events they experience, especially in the context of the war refugees. This study supports an alternative and original hypothesis about the role played by relational resilience in manifesting post-traumatic symptoms and promoting the recall and sharing (and potentially the processing) of stressful experiences. Future research could investigate how socio-cultural changes impact these processes over time, providing updates and comparisons with the current findings. Longitudinal studies, in particular, would offer a deeper understanding of the complex relationships between negative events, resilience, post-traumatic stress reactions, and memory over time.

## 5. Conclusions

This study highlights the central role of meaning-making and sharing in the natural relational contexts in which war refugee children and adolescents live. By increasing and harnessing the supportive capacities of these contexts, we can reactivate individuals’ epistemic trust, that is, their openness to others as reliable sources of old and new meanings with which to deal with the pain of the traumatic events they have witnessed. This support, contrary to what one might expect, can increase manifestations of post-traumatic stress, which are one of the ways in which the subject recalls memories and shares emotional feelings associated with traumatic events that had been patiently dissociated. Psychological interventions should therefore aim to reactivate these innate signifying capacities of human beings, both within therapeutic settings created for this purpose and in the natural contexts of children’s and adolescents’ lives. 

## Figures and Tables

**Table 1:** Spearman’s correlation coefficients between traumatic events, resilience, post-traumatic reactions, and event memories.

					Number of Memories (Timeline Activity)				
		CWTQ	CYRM-28	PTSR-C	Total	Positive	Negative	Trauma-Related	Trauma-Unrelated
CWTQ		—							
CYRM-28		0.095	—						
PTSR-C		0.354 ***	−0.067	—					
Number of Memories (Timeline Activity)	total	0.070	0.057	0.032	—				
	positive	0.010	0.146	−0.018	0.818 ***	—			
	negative	0.089	−0.054	−0.019	0.613 ***	0.111	—		
	trauma-related	0.357 ***	−0.182	0.340 ***	0.119	−0.078	0.363 ***	—	
	trauma-unrelated	−0.231 **	0.133	−0.308 ***	0.432 ***	0.191 *	0.525 ***	−0.456 ***	—

*Notes*. CWTQ = Child War Trauma Questionnaire; CYRM-28 = Childhood and Youth Resilience Measure-28; PTSR-C = Post-Traumatic Stress Reaction Checklist for Children; * *p* < 0.05, ** *p* < 0.01, *** *p* < 0.001.

**Table 2:** Parameters and estimates of the moderation model of each resilience subscales on the relationship between post-traumatic reactions and trauma-related memories (interaction effects).

	Estimate	SE	Z	Significance
PTSR-C ✻ CYRM_PSK	0.004	0.005	0.79	0.430
**PTSR-C ✻ CYRM_PSUP**	**0.016**	**0.008**	**1.98**	**0.048**
PTSR-C ✻ CYRM_IND_SSK	0.007	0.005	1.22	0.220
PTSR-C ✻ CYRM_FAM_PHY	0.018	0.012	1.50	0.140
**PTSR-C ✻ CYRM_FAM_PSY**	**0.011**	**0.005**	**1.99**	**0.047**
PTSR-C ✻ CRYM_SP	0.011	0.006	1.93	0.054
PTSR-C ✻ CYRM_ED	0.012	0.009	1.24	0.210
PTSR-C ✻ CYRM_CUL	0.004	0.005	0.71	0.480

*Notes*. PTSR-C = Post-Traumatic Stress Reaction Checklist for Children; CYRM = Child and Youth Resilience Measure; PSK = personal skills; PSUP = peer support; IND_SSK = individual’s social skills; FAM_PHY = physical support by family; FAM_PSY = psychological support by family; SP = spiritual resilience; ED = education; CUL = culture related resilience. Significant interactions are shown in bold (*p* < 0.05).

## Data Availability

The raw data supporting the conclusions of this article will be made available by the authors on request.
